# The psychological burden of major surgical complications in visceral surgery

**DOI:** 10.1007/s00423-024-03447-0

**Published:** 2024-08-20

**Authors:** Matthias Mehdorn, Helge Danker, Anne-Sophie Mehdorn

**Affiliations:** 1 Department of Visceral, Transplant, Thoracic and Vascular Surgery, University Hospital of Leipzig, Liebigstraße 20, 04103 Leipzig, Germany; 2 Department of Medical Psychology and Sociology, University of Leipzig, Leipzig, Germany; 3 Department of General, Abdominal, Transplantation, Thoracic and Pediatric Surgery, University Hospital Schleswig-Holstein, Campus Kiel, Germany

**Keywords:** Second victim, Psychological burden, Surgical complications, Coping mechanism

## Abstract

**Background:**

Complications are common after major visceral surgery. Besides the patients, also surgeons may experience negative feelings by the patients suffering. Some studies have evaluated the mental burden caused by complications, mainly focusing on residents in different surgical specialties. No evidence exists on the mental burden of board-qualified visceral surgeons in Germany.

**Materials and Methods:**

A point prevalence study was conducted using an online questionnaire. For the inclusion of participants, all departments of visceral surgery at German university hospitals were addressed. The objective of the online questionnaire was to elaborate the perception of complications and the coping mechanisms used by the surgeons with the aim to characterize the mental burden and possible improvement strategies.

**Results:**

A total of 113 questionnaires were answered, 98 being complete. 73.2% of the participants were male, 46.9% were consultants and had a working experience of 11–20 years. Most common specialties were colorectal and general surgery and 91.7% claimed to have caused complications Clavien-Dindo grade IV or V. Subsequently, predominant feelings were anger, grief, self-doubt and guilt. The fear of being blamed by colleagues or to lose reputation were high. Especially female and younger surgeons showed those fears. Coping mechanisms used to overcome those negative feelings were interaction with friends and family (60.6%) or proactive training (59.6%). Only 17.2% of the institutions offered professional support. In institutions where no support was offered, 71.6% of the surgeons asked for support.

**Conclusion:**

Surgical complications cause major psychological burden in surgeons in German university hospitals. Main coping mechanisms are communication with friends and families and professional education. Vulnerable subgroups, such as younger surgeons, may be at risk of suffering more from perceived mental distress. Nonetheless, the majority did not receive but asked for professional counselling. Thus, structured institutional support may ameliorate care for both surgeon and patient.

## Introduction

Visceral surgery is a challenging and demanding surgical specialty having to deal with different organs and tissues by oftentimes time consuming, surgically demanding and complex procedures. Complications are inherent in these treatments and range from simple wound infections, anastomotic leakages with necessity of surgical revision to life-threatening peritonitis or bleedings, potentially causing patients’ deaths. Complication severity is usually graded using the classification suggested by Clavien and Dindo [1]. Mortality rates of gastrointestinal surgery in Germany during the years 2009 until 2015 have been calculated to range from 6.9% (pancreas) to 8.6% (esophagus) and morbidity rates from 24.6% (colorectal) to 37.8% (esophagus) [2]. As complications occuring after surgery/postoperatively are classified surgical complications, not every surgical complication is surgeon-related, but have a multifactorial etiology. Certain risk factors may be modified preoperatively by the surgical staff, such as medication, physical [3] or nutritional [4] status, whilst others remain and add up to a patient specific surgical risk.

From a historic point of view, generations of surgeons have focused on technical aspects: They passed on the knowledge on how to sew the perfect anastomosis or to perfectly dissect in an anatomical plane in order to maximize the treatment’s benefits and reduce both surgical and medical complications. Yet, little effort has so far been put in on how to cope with one’s own complications mentally and the stress of being responsible for these complications, especially in elective surgery. In order to address this lack, the second victim theory has been developed advocating a major psychological burden of physicians who have unintentionally harmed their patients whilst treating them with the patient being the first victim of one procedure/complicative postoperative course and the surgeon the second victim [5]. The incapacity to deal with the psychological stress of a patient suffering from complications caused by oneself, may lead to burnout [6] or even suicidal tendencies [7], although surgeons might display higher psychological resilience than other people [8]. Nonetheless, high probability of burnout could be attributed to almost 50 percent of German surgeons in a survey, demonstrated by increased burnout scores [9].

Few studies have evaluated physician’s mental burden by complications in different medical specialties, often focusing primarily on young health care professionals [10, 11], evaluating their symptoms for mental distress. However, little is known about the mental burden and stress of highly skilled, board-qualified visceral surgeons performing complex elective procedures in German university hospitals. Furthermore, no evidence exists to what extent those surgeons would ask for professional support to deal with mental distress caused by their own surgical complications. Therefore, we conducted an online survey aiming to gain knowledge on the psychological burden of surgical complications on board-qualified, highly-specialized visceral surgeons in German university hospitals and their need of professional support.

## Materials and methods

Local ethics review board of the University Hospital of Leipzig was involved and due to the anonymized data the need for ethical approvement or informed consent was waived. The study was prospectively registered in the German register for clinical trials (DRKS00031451).

We performed a point prevalence survey using the online survey tool LimeSurvey which is provided by the institutional IT-department and hosted on local servers. Participating surgeons, including specialists, consultants, head of division or head of department, were recruited via email using institutional homepages of German university hospital departments of general and visceral surgery. As the study aimed at gaining a first idea of mental distress of visceral Surgeons in Germany, the cohort was limited to University hospitals only, as those hospitals presumably perform complex procedures at a high frequency. An invitation mail was sent including the link to the online survey. The survey was online for 30 days starting in May 2023.

An online questionnaire with a total of 16 questions was compiled, consisting of two blocks of questions, one on personal and one on professional circumstances. In order to reduce the required time for the questionnaire, a very limited number of questions was chosen. The aim of block one was to characterize the professional background of the participant, elucidating the surgeon’s main field of practice and the experience of complications Clavien-Dindo grade IV or V [1]. The second block precisely inquired about how participating surgeons perceived complications and in which way they dealt with them on a professional and emotional basis. The questionnaire consisted of yes/no questions, 10 item likert scale questions as well as multiple choice questions. The fears and sorrows that accompanied the complications were asked as 10-point likert scale from 1 (never) to 10 (always). The complete questionnaire is available in the supplementary materials.

After cessation of the online questionnaire the data was retrieved form the survey tool via Excel files and further analyzed with SPSS 29.0 (IBM statistics, Ehningen, Germany). Descriptive statistics were calculated for continuous variables (mean, standard deviation) and dichotomous variables. Univariate analyses of dichotomous variables were computed via cross tabs with X^2^-tests. Simple ANOVA was used for subgroup analyses of the likert-skales. P-values of less than 0.05 were considered significant.

## Results

### Demographics

A total of 767 mail addresses from all 35 German university hospital departments for General and Abdominal Surgery were used to send the invitation. The questionnaire was answered by 113 people, including 98 complete questionnaires, resulting in a response rate of 14.7 and 12.7% respectively. 73.5% of the participants were male. 38.9% were aged 30–40 years, 36.4% aged 40–50 years and 24.8% were older than 50 years. The majority (46.9%) had been working for 11–20 years, while 20.4% had been working for 6–10 years and 31% more than 20 years. 46.9% were consultants, 25.4% heads of surgical unit, 20.4% specialists and 7.1% heads of departments. General surgery and colorectal procedures were performed by 23.9% each. Hepatobiliary/transplantation (18.6%), upper gastrointestinaltract (13.3%) and pancreas procedures (8.8%) were less frequent.

The frequency of major surgeries was indicated as being performed on a daily base by 9.7%, three times per week by 39.8% and once per week by 29.2%.

### Complications

91.7% of the surgeons claimed to have caused or experienced complications Clavien-Dindo grade IV or V with 90.7% stating an emotional involvement in those complications. 85.4% felt guilty for the occurrence of the complication or the death of the patient.

**Fig. 1:**
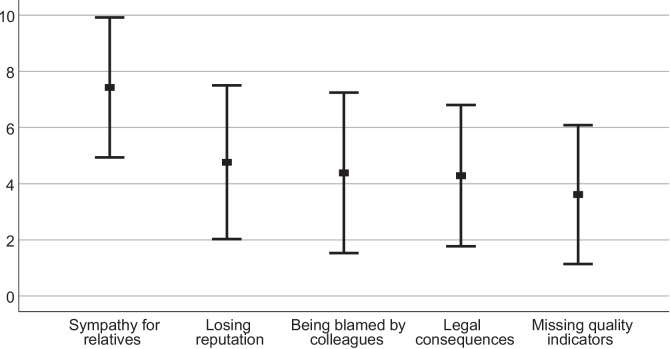
Fears in response of fatal complications. Values of answers on ten-point likert scales, expressed as means ± standard deviation

Sympathy for patients’ relatives was high (7.44 ± 2.85), whereas being accused by colleagues (4.39 ± 2.86), the fear of legal consequences (4.29 ± 2.52) or the fear of losing reputation (4.82 ± 2.74) was comparably lower (Fig. 1). Instead, the fear of attaining quality indicators (3.63 ± 2.47) yielded the lowest values in likert-skales.

**Fig. 2:**
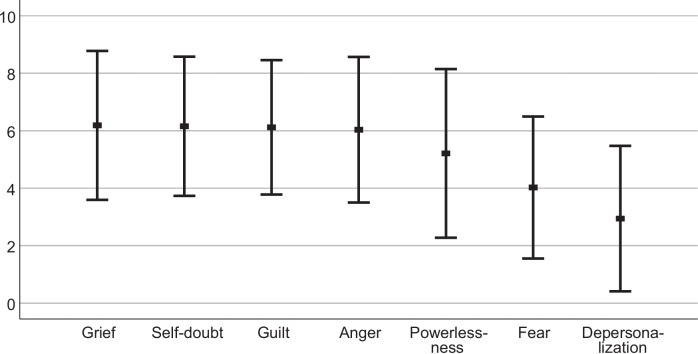
Feelings in response of fatal complications. Answers of ten-point likert scales expressed as means ± standard deviation

**Fig. 3:**
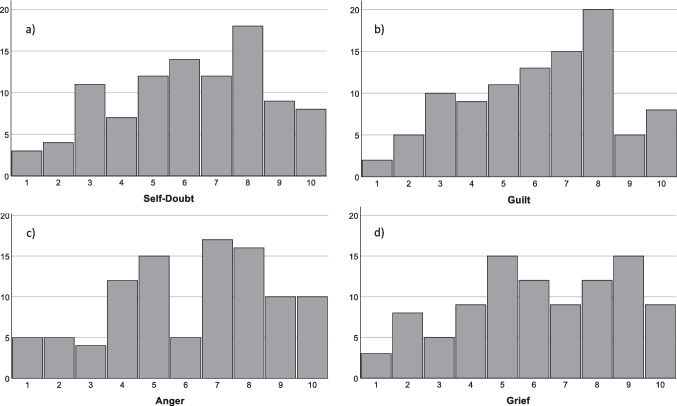
Exact histograms of answers on the ten-point likert scales depicting **a**) self-doubt, **b**) guilt, **c**) anger, **d**) grief. A and b show homogenous spread, c and d a two peaked pattern

Feelings of anger (6.23 ± 2.54), grief (6.2 ± 2.56), self-doubt (6.17 ± 2.42) and guilt (6.13 ± 2.35) were relatively high among the surgeons (Fig. 2). While self-doubt and guilt were homogenously spread along the scale with the mean at the respective score, the other two feelings showed a two-peaked response (Fig. 3). Powerlessness was concise, reaching a mean value of 5.35 ± 2.91. In contrast to that, severe mental reactions (depersonalization or derealization) were low (2.97 ± 2.50).

The analysis of mean values according to gender indicated some significant differences: Female surgeons had significantly more fears to be blamed by colleagues (5.33 ± 3.11 versus 4.03 ± 2.69; p = 0.043), to lose reputation (5.78 ± 2.65 versus 4.47 ± 2.7; P = 0.033) and the emotion of fear was generally more present compared to male colleagues (5.04 ± 2.69 versus 3.77 ± 2.43; p = 0.03). The same analysis carried out according to the position within the hierarchy showed significantly high values for heads of department being afraid of ruining quality indicators (6 ± 3.928 compared to 3.64 ± 2.35 for consultants; p = 0.024). Young specialists were significantly more afraid of being blamed (5.86 ± 2.46 compared to heads of units 3.27 ± 2.33; p = 0.27) and self- doubt was comparably more present but not statistically significant (7.14 ± 2.15 compared to consultants 5.89 ± 2.61). The only significant vocational experience-related parameter was the fear of being blamed (younger 5.85 ± 2.78 compared to more than 20 years of experience 3.68 ± 2.5; p = 0.023). Self- doubt was shown to be decreasing with experience, but not statistically significant.

### Coping mechanisms

**Fig. 4:**
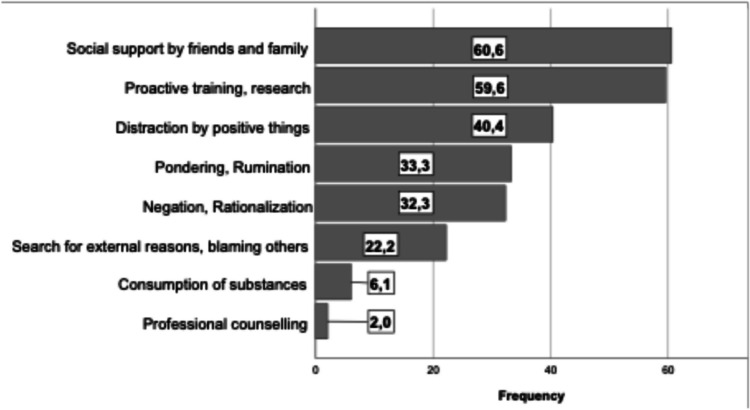
Coping mechanisms used by the participating surgeons. Values are given as percentages of the whole cohort. Participants could choose multiple answers

When being asked for their coping mechanisms in a multiple-choice question, most of the surgeons replied that they would cope with their mental distress searching for support within their social network (family and friends) or were looking to improve their skills via training or research, 60.6% and 59.6% respectively. 32.3% would negate or rationalize the events while 6.1% stated substance abuse. The whole list of responses is depicted in Fig. 4**.**

The analysis of coping mechanisms according to gender, experience or position revealed some significant results. The consumption of substances was stated by six consultants (one female, five male), four of those had been working 11–20 years. External attribution for the complication was stated by 22 surgeons, with consultants (p = 0.05) and those with 11–20 years of experience being underrepresented (p < 0.001) in this subgroup. The tendency to try to cope with complications by research or training was more present in more experienced surgeons while the interaction with friends and family was equally spread across the cohort.

### Professional coping

Routine mortality and morbidity conferences are held in most of the institutions (86.9%). Only 17.2% stated that the respective institution provided professional help for their employees to deal with critical incidents, which was not the case in 67.7% of the institutions, although 15.2% provided no information in this regard. Of those 17 people stating the professional counselling offers, only one person had used it. Only two male experienced surgeons stated professional counselling, thus one of them must have considered professional support privately. In contrast, of those with no possibility for professional support, 71.6% expressed the wish for professional support.

Using the X^2^-test to analyze if a certain group of surgeons expressed more often the wish for professional help, showed no differences in gender or position within the hierarchy. Although not statistically significant, colorectal surgeons and surgeons with 11–20 years of experience expressed more often the wish for professional support.

## Discussion

This point prevalence study showed, for the first time, the relevance of the psychological burden of fatal surgical complications on German board qualified, highly specialized visceral surgeons in German university hospitals.

### Study cohort

With the inclusion of board-qualified visceral surgeons from German university hospitals we aimed to provide data on German board qualified surgeons, including both consultants and specialists, in the context of increasing stress in daily surgical life. The study cohort represented a wide range of visceral surgery specialties, with colorectal and general surgery as main subspecialties. This is coherent to the frequency of those surgical procedures in which they are performed in Germany according to insurance data [2]. Upper GI and pancreatic surgery were a little less represented in our survey compared to the public data, as there are surgeons who perform different types of surgeries, but not only on one organ. With almost three quarters of invited and finally participating surgeons being male, this represents the structure found in most surgical departments in German university hospitals. Most of the replying colleagues were consultants with a medium time of experience in surgery (11–20 years). With more and more awareness for this topic, as well as more female medical students, it would be interesting to see how these results may vary in the near and long future.

### Emotional distress parameters

Emotional reactions to surgical adverse events are very common within various surgical specialties. Evidence is in line with our participants’ answers to have caused complications in life-threatening or nearly life-threatening manner [12]. The fear of being blamed for the complication or to lose reputation by those events was higher in female und inexperienced surgeons. As reported, people that have higher perceived stress, experience more emotional distress in exchange [8]. However, women are underrepresented in surgical departments, and therefore may be at a higher risk of experiencing emotional distress. In our study, negative influences diminished with working experience and were rather replaced by a prolific kind of coping, namely research and training. Women and men tend to deal differently with stressful situations in life, women more emotional based, men rather rational [13] which may explain the differences found between male and female surgeons. Except for the chair-people, our cohort was not worried about quality indicators. Furthermore, chair-people are known to have more resilience and experience less mental burden from their work [8], comparable to our findings. This may reflect special character traits a person should exhibit to be able to lead a surgical unit.

Nonetheless, we found comparably high values of guilt, anger and sadness [10–12, 14]. In those studies anxiety can be found in three quarters of surgeons [6, 10, 14] and guilt in 90% [10]. Two of those studies report the mental burden by residents. Therefore, inexperience with the situation, medically as well as mentally, was stressed as causative for the mental distress. Not only residents but also specialized surgical colleagues suffer from a relevant mental burden caused by severe complications after spine surgery [8], upper gastrointestinal tract [15] and colorectal surgery [14]. We report a collective of very experienced gastrointestinal surgeons that also exhibit relevant traits of those negative emotions. This illustrates that there may be some individuals more prone to those feelings than the other subgroup. The emotions reported in all of those studies as well as in ours represent signs of an acute stress disorder. Although we could not show a significant perception of depersonalization in our cohort, the emotions presented hint at a relevant emotional burden, increasing the risk for burnout. This would be coherent with literature that consultants, the largest group in our study, in surgery were most likely to show signs of burnout in the United Kingdom [16].

### Coping mechanisms for mental distress

In order to deal with mental distress and to prevent severe consequences such as burnout, different strategies can be used [13]. We report several strategies that may have helped the study participants to overcome the negative emotions evoked by the incidents. Most of them tried to cope in an emotional way (communication with family and friends, 60.6%) and professionally (research/ training, 59.6%). Still, negative coping by negation or pondering was also very common. As problem-focused coping, communicating with peers on a professional or emotional way has been described by others before [10–12, 17, 18] but the difference might be the respective group. In a professional environment one might not feel up to the task to reveal one’s feelings to a superior, especially if one belongs to a minority [19]. Our data shows that especially younger surgeons fear to be blamed, maybe by superiors in hierarchy. This would hinder a professional and trustworthy conversation with revelation of potential perceived weaknesses. If the counselling peer group would be at the same level of hierarchy, it will be easier to reveal oneself [10]. Generally, surgical departments in German university hospital are still very hierarchical. As residents form the largest group of surgeons in a department, the peer group shrinks in an upward direction in hierarchy. This might be reflected by the fact, that mostly those with an intermediate age in an intermediate hierarchical position (consultants) have the largest desire for counseling. Furthermore, this group may be experiencing the largest increase in responsibility paired with difficulties judging situations during the operation as surrogate of lack of experience [10, 14].

Self-distraction with positive things such as hobbies, also leading to emotion-based coping, was also very common. This kind of coping would rather be appreciated by common-sense, although there is evidence that emotion-focused coping is less effective and might lead to psychological distress [13]. Still, the combination of emotion-based coping together with distraction may be more effective than solely emotion-based coping.

It is remarkable that although all the participants in our study apparently had found a certain method of coping with the emotional distress after critical events, a large proportion (71.6%) expressed the wish for professional help to better deal with the emotional rollercoaster. We believe that this finding reflects a profound wish for communicating perceived distress by the events with a third party that is neither involved in private nor vocational matters. The findings are in line with data stated by the American pediatric surgeons [20]. In another survey in the UK and Ireland, about half of the surgeons expressed insufficient support by their institution, asking for different kinds of support [14]. Similar findings have been reported from Sweden where the lack of institutional support directly was linked to the psychological effects of being involved in the critical incident [18]. This lack of institutional help might simply be caused by a lack of advertising such programs [12]. The conclusion from Sweden was, in accordance with our findings, the request for more structured and direct follow-up which should be offered to healthcare professionals that have been involved in critical events.

### Improvement strategies

As our data as well as other studies show the need for and requested a structured system of offers for healthcare professionals to overcome critical events, what could be the distinct measures?

Several suggestions have been made: Recently, a standardized protocol was developed as conclusion of existing literature, consisting of the acronym PEARLS (Patient first, Emotional support, Apology, Review, Legal, Safety). The authors stress the importance of dealing with the harmed patient or the relatives but also to address emotional issues of the second victim [19]. Additionally, in another study a training program of peer supporters was established who would offer support to colleagues after a previously defined critical event [21]. A standardized questionnaire was used during the counselling sessions. The subjective evaluation within the department was an increase in “safety and support” culture. The counselling intervention triggered by a defined critical event facilitates access to supportive measures, especially for those who would not dare revealing themselves.

Those two examples may be just the beginning of a change in culture about coping in healthcare professionals as with second victims but the way to go is still very long.

This study has to deal with several limitations. First, only senior surgeons working at German University Hospitals were included in the study cohort. In the future, a similar survey may compare the distress between surgeons of different levels of different types of hospitals in order to gain a broader overview of the German surgical environment. Second, the response rate was only 14.7% and 12.7%, respectively. One might find the low number of female surgeons included astonishing. However, this represents the German surgical reality, with 23.5% female surgeons being among the board-qualified surgeons who were contacted via email. The study further has to deal with the limitations of a brief online questionnaire study, potentially lacking important questions regarding the topic. Therefore, this study can only show a trend regarding the opinion of German senior abdominal surgeons. Future research should be undertaken to compare the mental distress of surgeons working at different levels of hospitals, but also different specialities, to assess the mental distress more in detail and of course on improvement strategies.

## Conclusion

In our study we could demonstrate that highly specialized German visceral surgeons suffer from a significant mental distress caused by fatal complications. Although, at the moment, everyone copes in their own way, a large demand for professional support was brought up. A change in institutional support culture may ameliorate psychologic well-being of surgeons, which in the end will also help the patients.

## Data Availability

The data used in this study is available from the corresponding author upon reasonable request.
